# Virulence of Hungarian *Plasmopara halstedii* Isolates on Sunflower Differential Lines Carrying *Pl6*, *Pl8*, and *Pl_Arg_* Resistance Genes with Specific Instability of *Pl8-Mediated* Resistance

**DOI:** 10.3390/plants15091310

**Published:** 2026-04-24

**Authors:** Arbnora Berisha, Attila Kovács, Katalin Körösi, Ádám Ludányi, András Skornyik, Altin Berisha, Rita Bán

**Affiliations:** 1 Department of Integrated Plant Protection, Institute of Plant Protection, Hungarian University of Agriculture and Life Sciences, 2100 Godollo, Hungary; 2 Department of Agricultural and Environmental Engineering, University of Business and Technology, 10000 Pristina, Kosovo; 3 Syngenta, 1117 Budapest, Hungary; 4 Department of Livestock and Food Preservation Technology, Hungarian University of Agriculture and Life Sciences, 1118 Budapest, Hungary

**Keywords:** sunflower downy mildew, *Plasmopara halstedii*, virulence diversity, resistance genes, *Pl6*, *Pl8*, *Pl_Arg_*

## Abstract

Sunflower downy mildew, caused by *Plasmopara halstedii*, remains one of the most destructive diseases worldwide. The genetic diversity of *P. halstedii* populations continues to challenge resistance breeding efforts. This study evaluates the effectiveness of key resistance genes against *P. halstedii* isolates collected in Hungary. Eight isolates were tested using the sunflower differential lines HA-335, RHA-419, and RHA-340, with the resistance genes *Pl6*, *Pl_Arg_*, and *Pl8*, respectively. Disease development was assessed by observing sporulation and symptoms including stunting, chlorosis, damping-off, and abnormal development at three time points after inoculation. Plant height was also measured to evaluate growth responses. The *Pl6* resistance gene (HA-335) did not provide effective protection against any of the tested isolates, indicating that *Pl6* does not confer reliable resistance against the Hungarian isolates examined in this study. The resistance conferred by *Pl8* was not uniformly effective against the Hungarian isolates tested. This study provides the first report of *Pl8*-virulent *P. halstedii* isolates identified in both Hungary and Central Europe. The resistance gene *Pl_Arg_* (RHA-419) conferred resistance to all tested *P. halstedii* isolates. These findings highlight the changing virulence profiles of *P. halstedii* populations in Hungary, emphasizing the need for ongoing pathogen monitoring and strategic use of resistance genes.

## 1. Introduction

*Plasmopara halstedii* (Farl.) Berl. et de Toni, the causal agent of sunflower downy mildew, remains one of the most destructive biotic constraints to sunflower (*Helianthus annuus* L.) cultivation worldwide [1]. This obligate oomycete infects seedlings through the roots and rapidly colonizes host tissues systemically, resulting in stunting, chlorosis, abnormal leaf development, and severe yield losses under favorable conditions [2,3,4]. Downy mildew causes significant economic losses in major sunflower-producing regions, including Central and Eastern Europe [5,6]. In this context, an understanding of pathogen population structure and virulence evolution is essential for sustainable disease management.

A major challenge in sunflower downy mildew control is the high genetic diversity and rapid evolutionary potential of *P. halstedii* populations. Numerous studies have demonstrated that pathogen variability, driven by sexual recombination and long-distance dispersal, as well as by strong selection pressure from resistant cultivars, leads to the continuous emergence of new virulent pathotypes [7,8,9]. These dynamics undermine resistance strategies based on single major genes and complicate the development of durable resistance. Consequently, breeding for durable resistance demands detailed knowledge of host–pathogen interactions, isolate-specific virulence, and regional pathogen diversity.

Despite Hungary being one of Europe’s leading sunflower-producing countries, recent information on the virulence composition of *P. halstedii* populations is lacking. Earlier studies primarily focused on detecting highly virulent pathotypes and their spread, but comprehensive phenotypic evaluations of isolate aggressiveness and host responses have been scarce [6,10]. Over the past decades, extensive surveys across Europe have documented the emergence and spread of highly virulent *P. halstedii* pathotypes that can overcome key resistance genes, especially *Pl6* and *Pl8*, which historically formed the core of commercial sunflower resistance. Vear et al. [11] suggested that *Pl6* resistance may not correspond to a single gene conferring broad-spectrum resistance, but rather to a cluster of genes, each effective against one or a limited number of *P. halstedii* races. *Pl6* genes provided resistance to all pathotypes until the 2000s, but then virulent pathogen variants began to appear, first in France [12].

Subsequently, evidence of *Pl6* resistance erosion was reported from the USA in 2009 [13]. A later Europe-wide survey showed that more than half of the tested isolates were virulent on *Pl6*, including isolates from Spain, Portugal, Romania, Italy, France, and Hungary [6,14]. Resistance breakdown involving *Pl6* has now been widely reported across Europe, with increasing numbers of *Pl6*-virulent isolates detected in Central and Eastern European sunflower-growing regions [15,16].

The situation escalated further with the detection of *P. halstedii* isolates capable of overcoming *Pl8*, a resistance gene derived from *Helianthus argophyllus* and long regarded as one of the most effective resistance sources worldwide. During 2017–2018, severe downy mildew outbreaks were observed in commercial sunflower hybrids carrying *Pl8* in France and Italy, with disease incidences reaching 40–70% [17]. Inoculation assays on the traditional sunflower differential set confirmed that these isolates belonged to pathotype 714. Furthermore, it was virulent on RHA-340, a sunflower genotype carrying the *Pl8* resistance gene, which has not been included in the traditional differential set containing 9 or even 15 differential lines [3]. This was the first report of a breakdown in *Pl8* resistance in Europe. This finding confirmed that the currently used differential lines are not suitable for accurately estimating the virulence. Subsequent studies in the USA also demonstrated the occurrence of *Pl8*-virulent isolates [18].

In contrast to *Pl6* and *Pl8*, the resistance gene *Pl_Arg_*, also derived from *H. argophyllus*, has been reported to confer broad-spectrum resistance to a wide range of *P. halstedii* pathotypes and has attracted renewed attention as a potentially more durable resistance source. Fine-mapping and molecular characterization studies localized *Pl_Arg_* to linkage group 1 and identified resistance gene candidates associated with its function [19,20]. Marker-assisted selection and gene pyramiding strategies incorporating *Pl_Arg_* have been proposed as promising approaches to enhance resistance durability and mitigate the rapid erosion observed with single major genes [21,22,23]. Taken together, the rapid emergence of highly virulent *P. halstedii* pathotypes capable of overcoming *Pl6* and *Pl8* reflects an ongoing shift in the virulence profile of sunflower downy mildew. These changes threaten the prolonged effectiveness of major-gene resistance and accentuate the importance of continuous pathogen surveillance, expanded differential sets, and the strategic integration of novel resistance sources, including quantitative resistance and pyramided gene combinations. In this context, the present study addresses critical knowledge gaps by characterizing the virulence profiles of *P. halstedii* isolates collected from Hungary and evaluating the responses of sunflower genotypes HA-335, RHA-419, and RHA-340, which carry the resistance genes *Pl6*, *Pl_Arg_*, and *Pl8*, respectively. By combining disease severity assessments with plant growth responses and further symptom expressions, this work provides new data on local pathogen diversity and contributes to the development of durable resistance strategies for sustainable sunflower production.

## 2. Results

### 2.1. Disease Rates of Plasmopara halstedii Isolates: Assessment of Symptoms and Signs on Different Plant Genotypes

**Figure 1:**
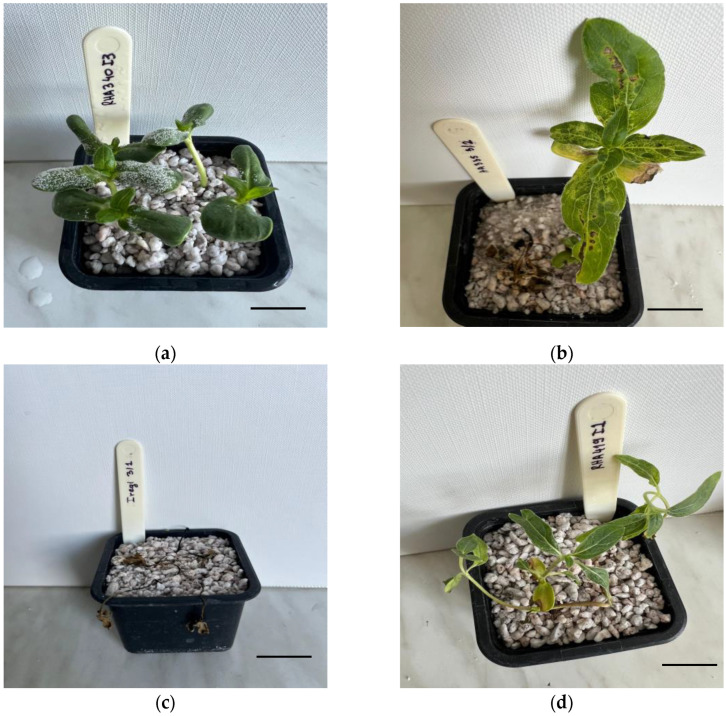
Symptoms and signs of sunflower downy mildew. (**a**) Sporulation of *P. halstedii* on cotyledons of RHA-340 (*Pl8*) following inoculation with isolate I3, visible as a white coating made of sporangiophores and sporangia. (**b**) Chlorosis and necrotic lesions on true leaves of HA-335 (*Pl6*) after inoculation with isolate 3/2. (**c**) Damping-off in the susceptible control genotype Iregi after inoculation with isolate 3/1. (**d**) Abnormal leaf development and growth distortion in RHA-419 (*Pl_Arg_*) following inoculation with isolate I1. Pictures are representative examples chosen to illustrate different symptom types and are not meant to show all isolate-genotype combinations. Scale bars represent 2 cm (**a**), 3 cm (**b**), 4 cm (**c**) and 4.5 cm (**d**).

Figure 1 shows the characteristic symptoms and signs of *P. halstedii* observed during the experiments. After inoculating young susceptible or partially resistant seedlings, the pathogen’s asexual reproductive organs (sporangiophores and sporangia) appeared as a white coating on the cotyledons (Figure 1a). Then, the pathogen systemically spread in susceptible plants, causing chlorotic symptoms (chlorosis) on the true leaves (Figure 1b). In many cases, susceptible plants died after germination or emergence, indicating typical damping-off symptoms (Figure 1c). Leaf curling and abnormal growth were also common symptoms in both susceptible and some resistant host–parasite interactions (Figure 1d).

**Figure 2:**
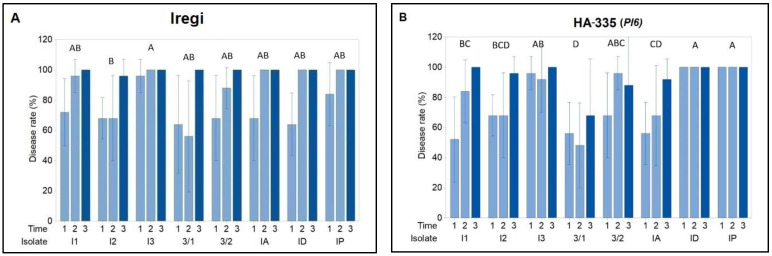

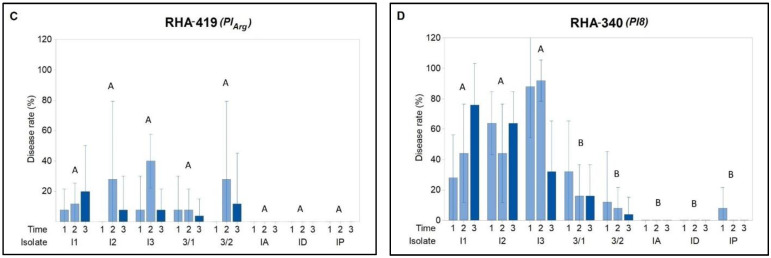
Disease rates (%) of sunflower genotypes (**A**) Iregi, (**B**) HA-335 (Pl6), (**C**) RHA-419 (Pl_Arg_), and (**D**) RHA-340 (Pl8) following inoculation with eight *Plasmopara halstedii* isolates (I1, I2, I3, 3/1, 3/2, IA, ID, and IP). Disease assessments were performed at three evaluation points: 1 = 9 days, 2 = 21 days, and 3 = 29 days post-inoculation. Bars represent mean values (*n* = 5), with standard deviations shown as error bars. Different letters above the central bars indicate significant differences in the average disease rates across the three evaluation times for the isolates.

Disease rates varied significantly among sunflower genotypes and *P. halstedii* isolates (Figure 2). The susceptible control cultivar Iregi showed consistently high disease rates across all isolates and assessment times, often exceeding 80%, which confirms its high inoculum viability and uniform infection pressure (Figure 2A). The infection rate of Iregi plants inoculated with the I2 *P. halstedii* isolate was slightly lower than the others, but it was only significantly different from that of plants infected with I3. In the HA-335 genotype carrying the *Pl6* gene, high disease rates were observed for most isolates, including I1, I2, I3, IA, ID, and IP, frequently approaching levels similar to the susceptible control (Figure 2B). Although isolates 3/1 and 3/2 caused moderately lower disease rates, infection increased at later assessment times. The RHA-419 sunflower genotype, which carries *Pl_Arg_*, exhibited a markedly different response pattern (Figure 2C). Disease progression was mostly limited for most isolates, with disease rates remaining close to zero for IA, ID, and IP throughout the assessment period. While some infection was observed with isolates I1, I2, I3, and 3/2, disease levels were much lower than in HA-335 and RHA-340. In genotype RHA-340 carrying *Pl8*, disease response varied by isolate (Figure 2D). Isolates I1, I2, and I3 caused moderate to high disease rates, whereas isolates 3/1 and 3/2 caused less infection. No disease was seen after inoculation with IA and ID, and only minimal infection occurred with IP.

### 2.2. Refining Symptom Assessment by Evaluating Plant Height, Damping-Off, Chlorosis, and Abnormal Plant Development

**Figure 3:**
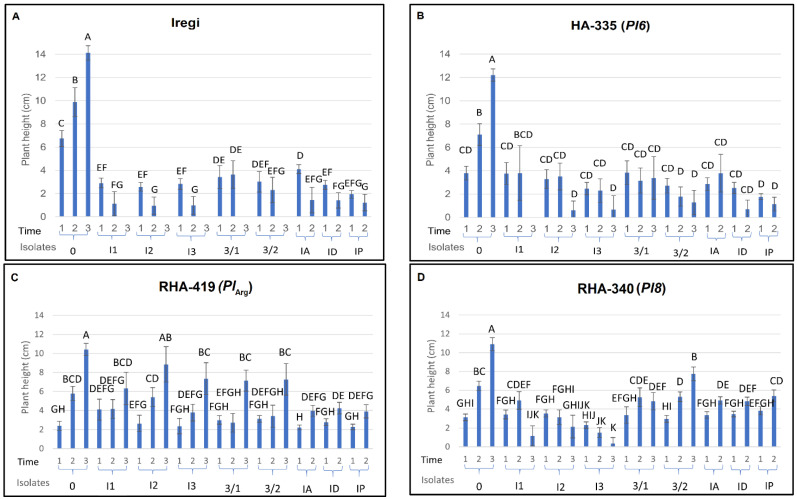
Plant height of sunflower genotypes: (**A**) Iregi (susceptible control), (**B**) HA-335 (Pl6), (**C**) RHA-419 (Pl_Arg_), and (**D**) RHA-340 (Pl8) after inoculation with eight *Plasmopara halstedii* isolates (I1, I2, I3, 3/1, 3/2, IA, ID, and IP). 0 indicates a non-inoculated control. Plant height was measured at three evaluation times (1 = 9, 2 = 21, and 3 = 29 days post-inoculation) for isolates I1, I2, I3, 3/1, and 3/2. For isolates IA, ID, and IP, height was assessed at two times (9 and 21 days post-inoculation). Plants inoculated with I1, I2, I3, 3/1, and 3/2 that showed a height of zero (e.g., Iregi and HA-335) had died from damping-off before the final evaluation. Values represent means (n = 25), with standard deviations as the error bars. Different letters indicate statistically significant differences among treatments within the same genotype (*p* < 0.05).

Plant height was significantly affected by genotype, isolate, and their interaction (Figure 3). In the susceptible control genotype Iregi, non-inoculated plants reached the greatest height, while inoculation with any *P. halstedii* isolate caused severe statistically significant growth suppression (Figure 3A). Height reduction was consistent across isolates and evaluation times, confirming the high aggressiveness of the isolates. Several isolate–genotype combinations (I1, I2, I3, 3/1, and 3/2) resulted in plant death due to damping-off by the third evaluation, resulting in a recorded height of zero. In the HA-335 genotype carrying *Pl6*, all isolates caused significant reductions in plant height compared to the non-inoculated control (Figure 3B). Growth suppression was especially notable after inoculation with isolates I3, 3/2, and IP. Plant height remained reduced at later assessment times. Conversely, inoculation of the genotype RHA-419 carrying *Pl_Arg_* with different *P. halstedii* isolates caused limited reductions in height (Figure 3C). Additionally, there was no significant difference between plants inoculated with the I2 isolate and non-inoculated plants. However, the genotype RHA-340 carrying *Pl8* displayed pronounced isolate-dependent growth responses (Figure 3D). Isolates I1, I2, I3, and 3/1 caused strong suppression of plant height, while growth reduction was less evident following inoculation with isolate 3/2. For isolates IA, ID, and IP, plant height was only measured at the first two evaluation times, and these *P. halstedii* isolates induced moderate growth suppression in the RHA-340 genotype.

Distinct genotype- and isolate-dependent differences were observed in damping-off incidence, chlorosis, and abnormal leaf development following inoculation with *P. halstedii* isolates (Table 1). In the susceptible control cultivar Iregi, damping-off reached 100% for isolates I1, I2, I3, 3/1, and 3/2 when assessed at the third evaluation, confirming extreme susceptibility and severe early infection. For isolates IA, ID, and IP, the damping-off percentages were lower, ranging from 52% to 68%. This was observed during the second evaluation, indicating that the disease’s progression varied among the isolates. Chlorosis was detected at low to moderate levels depending on the isolate, while abnormal leaf development was not observed in the Iregi cultivar.

In the HA-335 genotype carrying the *Pl6* gene, high damping-off rates were observed across all isolates (Table 1). Complete seedling collapse occurred after inoculation with isolates I1 and I3 at the third evaluation, while isolates IA, ID, and IP showed reduced but still significant damping-off (28–72%) during the second evaluation. Abnormal leaf development was not observed in HA-335, likely due to early seedling mortality, which limited the appearance of further symptoms.

In contrast, the RHA-419 genotype carrying the *Pl_Arg_* gene showed significantly less damping-off across most isolates (Table 1). However, the level of damping-off observed in RHA-419 plants inoculated with isolates I1 and 3/2 is notable, at 16% and 12%, respectively. Chlorosis was not seen in any of the *Pl_Arg_* interactions. Additionally, abnormal leaf development was consistently observed with isolates I3, 3/1, and 3/2.

The RHA-340 genotype, which carries the *Pl8* gene, showed distinct responses across isolates (Table 1). High damping-off and chlorosis were observed after inoculation with isolates I1, I2, and I3 during the third evaluation. In contrast, isolates 3/1 and 3/2 caused very little damping-off but were linked to significant abnormal leaf development. For isolates IA, ID, and IP, no damping-off, chlorosis, or abnormal leaf symptoms were seen at the second evaluation.

### 2.3. Virulence of Plasmopara halstedii Isolates on Plant Genotypes with Different Resistance Genes

Summarizing the results from disease rates, plant heights, damping-off, chlorosis, and abnormal leaves, the virulence of eight *P. halstedii* isolates were evaluated on four sunflower genotypes with different resistance backgrounds (Table 2). The control cultivar, Iregi, was shown to be susceptible to all isolates. Likewise, the HA-335 genotype carrying *Pl6* was susceptible to all tested isolates, indicating that Pl6 did not confer effective resistance to the *P. halstedii* isolates studied. In contrast, the RHA-419 genotype, which carries *Pl_Arg_,* demonstrated resistance to all eight isolates, with no compatible interactions observed. However, further quantitative assessments revealed isolate-dependent variation in disease severity and plant growth effects. The RHA-340 genotype, carrying *Pl8*, showed reactions specific to certain isolates. After inoculation with isolates I1, I2, and I3, susceptibility was observed, while resistant responses were observed with isolates 3/1, 3/2, IA, ID, and IP. Overall, the pathogenicity tests highlight clear differences in resistance effectiveness among *Pl6*, *Pl8*, and *Pl_Arg_*.

## 3. Discussion

The current study shows significant variation in the virulence of *P. halstedii* isolates collected from Hungary over the past five years. All isolates infected the susceptible control cultivar Iregi and consistently exhibited high disease incidences, severe damping-off, and strong growth suppression. This consistent susceptibility confirms the effectiveness of the inoculation method, verifies isolate viability and aggressiveness, and provides a reliable baseline for assessing resistance responses in plant genotypes with different resistance genes.

Building on our findings, the *Pl6* resistance gene carried by HA-335 did not provide effective protection against any of the tested isolates, indicating that *Pl6* does not confer reliable resistance against the Hungarian isolates examined in this study. This conclusion is supported by multiple indicators: high disease rates, extensive damping-off, frequent chlorosis, and ongoing growth suppression. In practice, these results suggest that *Pl6* did not reduce the damaging effects of infection on plant growth. These observations align with previous reports of *Pl6* resistance breaking down across Europe and beyond [6,13,24]. The historical reliance on *Pl6*-based hybrids likely created strong selection pressure on *P. halstedii* populations, promoting the emergence and spread of virulent pathotypes [9].

It should be noted that pooling disease assessments across time points does not account for the temporal autocorrelation inherent to systemic infection processes. Therefore, the statistical results primarily reflect overall differences in isolate aggressiveness rather than detailed temporal dynamics of disease progression. Future studies should employ repeated-measures or mixed-effects models to better capture disease development over time.

In contrast to *Pl6*, *Pl8*-mediated resistance showed a variable response that largely depended on the isolate’s identity. The RHA-340 line, which carries the *Pl8* gene, was clearly susceptible to isolates I1, I2, and I3 but showed resistance to isolates 3/1, 3/2, IA, ID, and IP. This isolate-specific behavior was consistent across disease-rate data, damping-off incidence, chlorosis development, and plant-height responses, indicating that *Pl8* resistance is not reliably effective against the Hungarian isolates tested. Notably, the susceptibility of RHA-340 to isolates I1, I2, and I3 provides evidence of the presence of *Pl8*-virulent *P. halstedii* isolates in Hungary. To our knowledge, this is the first report of *Pl8*-virulent *P. halstedii* isolates in both Hungary and Central Europe. At the population level, our findings suggest a partial breakdown of the *Pl8* resistance gene, making this an important discovery for guiding national resistance deployment and breeding strategies. Resistance conferred by major *Pl* genes, including *Pl8*, is generally consistent with a gene-for-gene interaction model. However, the molecular basis of Pl8-mediated resistance and the identity of *P. halstedii* effectors capable of overcoming this resistance remain largely uncharacterized [3]. This lack of molecular information may contribute to the observed variability in resistance responses and highlights the need for further research.

In the European context, *Pl8* breakdown was first confirmed in France and Italy [17] and has since been recognized as a major threat to breeding programs, with further confirmation of *Pl8*-virulent populations in other regions, including North America [18]. The present results extend this pattern by showing that *Pl8*-virulent isolates are also found in Hungary. This is significant because commercial sunflower hybrids containing *Pl8* are common in Europe and the USA [17,18].

It is well known that the traditional, internationally accepted differential set used to identify sunflower downy mildew pathotypes contains nine sunflower lines with different resistance genes [25]. One of the most advanced resistance genes in the set is *Pl6*. Tourvieille de Labrouhe et al. [12] proposed expanding the set by including sunflower lines with additional resistance genes, such as *Pl_Arg_*. However, this expanded system still does not contain important genes used in sunflower breeding, such as *Pl8;* hence, the same nominal pathotype designation may mask biologically meaningful virulence differences [17]. Therefore, the lack of a differential line with the *Pl8* gene in the classical set, as well as the set’s known limitations, and the rapid spread of highly virulent pathotypes highlight the need to revise and expand pathotype identification frameworks [15,26,27].

Among the resistance genes evaluated, *Pl_Arg_* (RHA-419) showed the highest effectiveness, which demonstrated resistance to all tested *P. halstedii* isolates. This supports reports identifying *Pl_Arg_* as a broadly effective resistance source in *H. agrophyllus* [19,20,21]. *Pl_Arg_* has provided reliable resistance to various pathotypes of *P. halstedii* for several decades in sunflower-growing regions around the world [18,27,28,29,30,31]. However, it is noteworthy that isolates I1 and 3/2 caused damping-off in more than 10% of cases, while isolates I3, 3/1, and 3/2 resulted in abnormal leaf development in more than 30% of cases.

Nevertheless, these observations on genotypes carrying *Pl_Arg_* should be interpreted with caution. Although most plants remained phenotypically healthy at the final evaluation and showed no systemic disease symptoms, the presence of abnormal leaf development may indicate a more complex host–pathogen interaction. This response could reflect either a localized defense reaction or a limited level of pathogen colonization that cannot be resolved based on macroscopic symptoms alone. Therefore, these findings may represent an early signal of partial pathogenicity or incomplete resistance rather than a purely physiological growth-related effect. Further microscopic and histological analyses would be required to distinguish between a hypersensitive response and restricted infection.

Additionally, the quantitative data may demonstrate that *Pl_Arg_* resistance is not necessarily “cost-free”. Although the development of systemic disease was restricted (e.g., no chlorosis was recorded), certain isolate–genotype combinations produced mild disease symptoms, with moderate reductions in plant height. *Pl_Arg_* restricted pathogen colonization and/or systemic development rather than fully preventing early infection. Such partial resistance could contribute to improved durability by reducing selection pressure on pathogen populations, but it can also entail physiological costs or developmental penalties. In this context, defense-related trade-offs between growth and resistance have been described in other host–pathogen systems [32,33], and the *Pl_Arg_* responses observed here are consistent with that general framework.

It should be noted that plant height values include zero measurements corresponding to dead plants (damping-off), thereby integrating both survival and growth responses. In several cases, complete mortality was observed, while in others, only a small number of plants survived, making height measurements from surviving individuals alone unrepresentative. Therefore, plant height should be interpreted as an integrated measure of disease impact rather than growth of surviving plants only.

Collectively, these results show that *P. halstedii* populations in Hungary possess virulence profiles that can overcome widely deployed resistance genes. The complete loss of *Pl6* effectiveness and the isolate-dependent erosion of *Pl8* highlight the vulnerability of relying on single major genes under sustained selection pressure. Importantly, the detection of *Pl8*-virulent isolates in Hungary complements earlier Hungarian observations and monitoring efforts reporting high-virulence pathotypes and the evolving nature of national pathogen populations [1,6,34]. By integrating virulence characterization with quantitative measures of symptoms and growth, the present study strengthens the evidence base for updating resistance deployment strategies in sunflower.

From a breeding perspective, the results support diversifying resistance sources as priorities for durable downy mildew management. *Pl_Arg_* remains a highly valuable resistance component, but its partial nature and the presence of isolate-dependent developmental effects suggest it should not be deployed in isolation. Marker-assisted selection and gene pyramiding strategies offer practical routes to combine *Pl_Arg_* with additional major or quantitative loci to enhance durability [22,23]. In parallel, continued monitoring of *P. halstedii* populations and systematic characterization of isolates across regions remain essential to anticipate shifts in virulence and proactively adapt breeding strategies. Overall, this study provides new, isolate-resolved evidence of resistance erosion in Hungary—especially with respect to *Pl8*—and supports integrated resistance breeding as a cornerstone of sustainable sunflower production under an evolving pathogen threat.

## 4. Materials and Methods

### 4.1. Sunflower Genotypes and Plasmopara halstedii Isolates

Four sunflower genotypes were used in this study: Iregi szürke csíkos (Iregi), HA-335, RHA-419, and RHA-340. The Hungarian cultivar Iregi, which is susceptible to all known pathotypes of *P. halstedii*, was used as a susceptible control and as a host for increasing inoculum quantity. The sunflower inbred lines HA-335, RHA-419, and RHA-340 were selected as differential genotypes for resistance evaluation, which carry resistance genes *Pl6, Pl_Arg,_* and *Pl8,* respectively. A total of eight *P. halstedii* isolates were included in the experiments, which were collected in Hungary in 2021, 2024, and 2025 (Table 3). The origin of the isolates and their associated host resistance backgrounds were determined based on the commercial sunflower hybrids from which they were collected. All isolates were maintained on infected sunflower tissue in an ultra-low temperature freezer at −70 °C to preserve viability until use.

### 4.2. Preparation of Plasmopara halstedii Inoculum and Inoculation

For inoculum preparation and inoculation, we used the method described by Trojanová et al. [26]. Briefly, seeds of Iregi, HA-335, RHA-419, and RHA-340 were surface-sterilized in 1% NaOCl for 3–5 min to eliminate surface contaminants, and then thoroughly rinsed with running tap water. Seeds were germinated on moist filter paper under controlled conditions. Based on the germination vigor of the sunflower genotypes, germination was carried out at 19 °C for Iregi and at 24 °C for HA-335, RHA-419, and RHA-340 for 2–3 days, until radicles reached 3–5 mm.

Sporangial suspensions were prepared from infected leaf tissues of preserved isolates by gently rinsing the tissue in bidistilled water. Inoculation was performed using the whole seedling immersion (WSI) [35] as follows: germinated seeds were first rinsed in bidistilled water for 5–6 min. Infected leaf material of each isolate was removed from cold storage, and sporangia were released by gently brushing the leaf surface in 100 mL of bidistilled water. Then the sporangial concentration was adjusted to 50,000 sporangia mL^−1^ using a Bürker counting chamber. For each isolate–genotype combination, approximately 25 mL of sporangial suspension was used to ensure complete immersion of all seedlings during inoculation. Seeds were transferred to Petri dishes for each isolate-genotype combination. Control dishes contained an equal number of seeds immersed in bidistilled water. Seeds were fully submerged in the sporangial suspension and incubated overnight in darkness at 16 °C to promote infection.

### 4.3. Plant Cultivation

After inoculation, seedlings were transplanted into pots (10 pots per isolate-genotype combination, 5 seedlings per pot) containing horticultural perlite (d = 4 mm). Pots were placed in trays and cultivated in a growth chamber under a 12-h photoperiod at 22 °C and under a light intensity of 100 μE·m^−2^·s^−1^. Plants were watered every two days. A nutrient solution (Volldünger Linz, 0.2 g L^−1^, 0.5 L per tray) was applied three days after sowing to support seedling development.

### 4.4. Sporulation Induction and Disease Assessments

Sporulation was induced 9 days post-inoculation (dpi). Trays were placed in dark plastic bags, and plants were sprayed with bidistilled water before the bags were sealed, creating high-humidity conditions favorable for pathogen development. The sealed trays were incubated in darkness at 19 °C for 24 h. We performed disease assessments three times: 1. at 10 dpi, right after sporulation; 2. at 21 dpi, when most plants had developed true leaves, allowing for the evaluation of systemic infection; and 3. at 29 dpi. Plants were classified as healthy or diseased based on the absence or appearance of visible symptoms, including sporulation on cotyledons, chlorosis on true leaves, abnormal plant development (stunting, curling, asymmetric true leaves), and damping-off. If only slight sporulation was observed on the plants without the appearance of further symptoms, the reaction was assessed as resistant. The disease rate (%) was calculated as the percentage of diseased plants. Because downy mildew infection causes pronounced growth suppression, plant height was measured at 10, 21, and 29 dpi.

### 4.5. Statistical Analysis

The experimental unit for disease-related parameters was the pot, with each pot containing five seedlings. Disease rate was calculated as the mean value per pot (n = 5 pots per isolate–genotype combination). For plant height measurements, individual plants were used as statistical units. However, plants within the same pot may not be fully independent due to shared microenvironmental conditions.

Two independent experiments were conducted. Statistical analyses were performed using IBM SPSS Statistics (version 25). Assuming normal distributions because of the relatively large sample size (n = 15–25 for disease assessments and 50–75 for plant height measurements per genotype-isolate combinations), homogeneity of variances was assessed using Bartlett’s test. Since equal variance was not satisfied in either case, we used Welch-Anova with the Games-Howell Pairwise Comparisons as a post hoc test (*p* ≤ 0.05) [36].

Because the number of replicates (n = 5) per individual evaluation time was insufficient to assess distributional assumptions and perform robust parametric comparisons, statistical analyses were not conducted separately for time points 1, 2, and 3 during disease assessment. To increase statistical power and meet the assumptions required for parametric testing, disease rate data were pooled across all three assessment periods (total n = 15 per isolate–genotype combination). The larger combined sample size allowed for the assumption of approximate normality and enabled statistical comparisons among isolates [36].

Disease progression over time (9, 21, and 29 dpi) was evaluated descriptively, and no formal statistical comparison across time points or time × isolate interactions was performed due to the limited number of replicates per time point. Disease rate data from multiple assessment time points represent repeated measurements on the same experimental units and are therefore not independent; consequently, the pooled analysis should be interpreted as an exploratory approach and does not fully account for temporal dependence. Disease reactions were additionally interpreted using threshold-based criteria, where values above 60% were considered susceptible and values below 20% resistant.

## 5. Conclusions

This study shows significant differences in virulence among *P. halstedii* isolates collected in Hungary over the last 5 years. It highlights contrasting resistance profiles of the major sunflower downy mildew resistance genes. The *Pl6* gene no longer provides effective protection against most tested isolates, while *Pl8* resistance was selectively overcome across isolates, confirming the ongoing weakening of widely used resistance sources. In contrast, the *Pl_Arg_* gene conferred the highest level of resistance, substantially reducing disease development and early seedling damping-off. However, minor growth and morphological effects suggest a partial resistance response. These findings highlight the limitations of relying on a single dominant resistance gene and emphasize the need to incorporate durable resistance strategies, including gene pyramiding, quantitative resistance, and ongoing pathogen monitoring. A better understanding of local *P. halstedii* virulence patterns will be crucial for guiding sunflower breeding programs toward more sustainable and long-term disease management.

## Figures and Tables

**Table 1:** Incidence of damping-off, chlorosis, and abnormal leaf development (mean ± SD, %) in sunflower genotypes Iregi, HA-335 (*Pl6*), RHA-419 (*Pl_Arg_*), and RHA-340 (*Pl8*) after inoculation with eight *Plasmopara halstedii* isolates.

Isolate	Plant Genotype (Resistance Gene)	Damping-Off (Mean ± SD)	Chlorosis (Mean ± SD)	Abnormal Leaf (Mean ± SD)
I1	Iregi HA-335 (*Pl6*) RHA-419 (*Pl_Arg_*) RHA-340 (*Pl8*)	**100** ± 0.0 **100** ± 0.0 16 ± 8.4 **64** ± 20.5	16 ± 13 8 ± 5.5 0.0 0.0	0.0 0.0 0.0 0.0
I2	Iregi HA-335 (*Pl6*) RHA-419 (*Pl_Arg_*) RHA-340 (*Pl8*)	**100** ± 0.0 **84** ± 13.0 8 ± 8.9 **48** ± 15.1	16 ± 10.10 8 ± 9 0.0 0.0	0.0 0.0 0.0 0.0
I3	Iregi HA-335 (*Pl6*) RHA-419 (*Pl_Arg_*) RHA-340 (*Pl8*)	**100** ± 0.0 **100** ± 0.0 8 ± 5.5 **92** ± 5.5	4 ± 4.5 **24** ± 13 0.0 **32** ± 16.7	0.0 0.0 **36** ± 10.9 0.0
3/1	Iregi HA-335 (*Pl6*) RHA-419 (*Pl_Arg_*) RHA-340 (*Pl8*)	**100** ± 0 **68** ± 15 0.0 8 ± 9	8 ± 8.9 8 ± 5.5 0.0 4 ± 4.5	0.0 0.0 **32** ± 11.4 16 ± 8.3
3/2	Iregi HA-335 (*Pl6*) RHA-419 (*Pl_Arg_*) RHA-340 (*Pl8*)	**100** ± 0.0 **64** ± 13.0 12 ± 13.4 4 ± 4.7	**24** ± 16.4 **36** ± 8.4 0.0 0.0	0.0 0.0 **36** ± 21.6 **40** ± 20
IA	Iregi HA-335 (*Pl6*) RHA-419 (*Pl_Arg_*) RHA-340 (*Pl8*)	68 ± 16.7 * 28 ± 5.5 * 0.0 * 0.0 *	8 ± 8.9 20 ± 7.1 0.0 0.0	0.0 0.0 0.0 0.0
ID	Iregi HA-335 (*Pl6*) RHA-419 (*Pl_Arg_*) RHA-340 (*Pl8*)	**52** ± 8.9 * 72 ± 8.9 * 0.0 * 0.0 *	0.0 8 ± 8.9 0.0 0.0	0.0 0.0 0.0 0.0
IP	Iregi HA-335 (*Pl6*) RHA-419 (*Pl_Arg_*) RHA-340 (*Pl8*)	**64** ± 13 * 52 ± 19.5 * 0.0 * 0.0 *	0.0 4 ± 4.5 0.0 0.0	0.0 0.0 0.0 0.0

* For isolates IA, ID, and IP, damping-off values were recorded during the second evaluation (21 days post-inoculation) because these isolates were not included in the third evaluation. For all other isolates, damping-off was assessed at the third evaluation (29 days post-inoculation).

**Table 2:** Virulence of eight *Plasmopara halstedii* isolates (I1, I2, I3, 3/1, 3/2, IA, ID, and IP) on sunflower genotypes Iregi, HA-335 (*Pl6*), RHA-419 (*Pl_Arg_*), and RHA-340 (*Pl8*). Reactions were classified as resistant (R) or susceptible (S) based on the presence or absence of pathogen sporulation on cotyledons and leaves, damping-off, chlorosis on true leaves, and abnormal development.

Isolate	Genotype	Reaction R/S
I1	Iregi HA-335 (*Pl6*) RHA-419 (*Pl_Arg_*) RHA-340 (*Pl8*)	**S** **S** R **S**
I2	Iregi HA-335 (*Pl6*) RHA-419 (*Pl_Arg_*) RHA-340 (*Pl8*)	**S** S R **S**
I3	Iregi HA-335 (*Pl6*) RHA-419 (*Pl_Arg_*) RHA-340 (*Pl8*)	**S** **S** R **S**
3/1	Iregi HA-335 (*Pl6*) RHA-419 (*Pl_Arg_*) RHA-340 (*Pl8*)	**S** **S** R R
3/2	Iregi HA-335 (*Pl6*) RHA-419 (*Pl_Arg_*) RHA-340 (*Pl8*)	**S** **S** R R
IA	Iregi HA-335 (*Pl6*) RHA-419 (*Pl_Arg_*) RHA-340 (*Pl8*)	S **S** R R
ID	Iregi HA-335 (*Pl6*) RHA-419 (*Pl_Arg_*) RHA-340 (*Pl8*)	**S** **S** R R
IP	Iregi HA-335 (*Pl6*) RHA-419 (*Pl_Arg_*) RHA-340 (*Pl8*)	**S** **S** R R

**Table 3:** Origin of *Plasmopara halstedii* isolates used in this survey.

Isolate	Year of Collection	Place of Origin	Host Resistance Gene ^1^
I1	2021	Hungary	*Pl6*
I2	2021	Hungary	*Pl6*
I3	2021	Hungary	*Pl6*
3/1	2025	Hungary	*Pl6*
3/2	2025	Hungary	*Pl6*
IA	2024	Hungary	*Pl6*/*Pl7*
ID	2024	Hungary	*Pl6*/*Pl7*
IP	2024	Hungary	*Pl6*/*Pl7*

^1^ Host resistance gene refers to the resistance gene present in the sunflower genotype against *Plasmopara halstedii.*

## Data Availability

The original contributions presented in this study are included in the article. Further inquiries can be directed to the corresponding author.
